# Changes in Food Choices of Participants in the Special Diabetes Program for Indians–Diabetes Prevention Demonstration Project, 2006–2010

**DOI:** 10.5888/pcd12.150266

**Published:** 2015-11-12

**Authors:** Nicolette I. Teufel-Shone, Luohua Jiang, Janette Beals, William G. Henderson, Kelly J. Acton, Yvette Roubideaux, Spero M. Manson

**Affiliations:** Author Affiliations: Luohua Jiang, University of California, Irvine, Irvine, California; Janette Beals, Spero M. Manson, Centers for American Indian and Alaska Native Health, University of Colorado Anschutz Medical Center, Aurora, Colorado; William G. Henderson, Colorado Health Outcomes Program, University of Colorado Anschutz Medical Center, Aurora, Colorado; Kelly J. Acton, US Department of Health and Human Services, San Francisco, California; Yvette Roubideaux, Indian Health Service, Rockville, Maryland.

## Abstract

**Introduction:**

American Indians/Alaska Natives (AI/ANs) have a disproportionately high rate of type 2 diabetes. Changing food choices plays a key role in preventing diabetes. This study documented changes in the food choices of AI/ANs with diagnosed prediabetes who participated in a diabetes prevention program.

**Methods:**

The Special Diabetes Program for Indians–Diabetes Prevention Demonstration Project implemented the evidence-based Diabetes Prevention Program (DPP) lifestyle intervention in 36 health care programs nationwide, engaging 80 AI/AN communities. At baseline, at 30 days post-curriculum, and at the first annual assessment, participants completed a sociodemographic survey and 27-item food frequency questionnaire and underwent a medical examination assessing fasting blood glucose (FBG), blood pressure, body mass index (BMI), low-density lipoprotein [LDL], high-density lipoprotein [HDL], and triglycerides. Multiple linear regressions were used to assess the relationship between temporal changes in food choice and other diabetes risk factors.

**Results:**

From January 2006 to July 2010, baseline, post-curriculum, and first annual assessments were completed by 3,135 (100%), 2,046 (65%), and 1,480 (47%) participants, respectively. An increase in healthy food choices was associated initially with reduced bodyweight, BMI, FBG, and LDL and increased physical activity. At first annual assessment, the associations persisted between healthy food choices and bodyweight, BMI, and physical activity.

**Conclusion:**

AI/AN adults from various tribal and urban communities participating in this preventive intervention made sustained changes in food choices and had reductions in diabetes risk factors. The outcomes demonstrate the feasibility and effectiveness of translating the DPP lifestyle intervention to community-based settings.

## Introduction

From 1994 to 2009, the prevalence of diagnosed type 2 diabetes increased by 161% among American Indian and Alaska Native (AI/AN) adults aged 25 to 34 (1). To reverse this trend, addressing diabetes risk factors is paramount. In clinical trials, achieving moderate weight loss and maintaining blood pressure (BP), fasting blood glucose (FBG) and blood lipids (high-density lipoprotein [HDL], low-density lipoprotein [LDL], and triglycerides) within recommended ranges through lifestyle intervention delays and prevents diabetes onset and related cardiovascular complications (2–4). People at risk for diabetes who modify their lifestyle are more likely to sustain long-term changes in food choices than to adhere to recommended levels of physical activity (5,6). Thus developing new food habits plays a key role in the sustained prevention of diabetes. Changes in diet, particularly increased intake of low-fat, high-fiber foods, play a key role in prevention (7) and are associated with decreased bodyweight, reduced glycosylated hemoglobin (HbA1C), and increased insulin sensitivity (8–10). The impact of changes in food choice is particularly striking in high-risk populations such as AI/ANs, Hispanics, and African Americans (10,11). Translating evidence-based lifestyle interventions to real-world settings and resources of populations that have a disproportionate share of diabetes can decrease the incidence of diabetes (1).

In 2004, the Indian Health Service (IHS) (12) invited tribal and urban Indian health programs and IHS Service Units to apply for competitive funding to assess the feasibility of using proven diabetes prevention strategies in AI/AN communities. The resulting Special Diabetes Program for Indians–Diabetes Prevention (SDPI–DP) Demonstration Project adapted and implemented the lifestyle intervention developed by the Diabetes Prevention Program (DPP), a multicenter randomized clinical trial sponsored by the National Institute of Diabetes and Digestive and Kidney Diseases (3). Although the DPP included a small number of AI/ANs (n = 161), it was a research trial implemented in well-controlled settings. The SDPI–DP project translated the findings of the DPP clinical trial into a program for culturally and geographically diverse AI/AN communities and assessed its feasibility and effectiveness (12). In the present study, site-specific data were aggregated to describe changes in food choices of participants in the SDPI–DP project from 2006 to 2010. 

## Methods

### Participant population

The SDPI–DP project is being conducted in 11 of 12 IHS administrative areas nationally and represents tribal, urban, and federally administered programs, serving 80 tribes in 18 states. The University of Colorado Anschutz Medical Center serves as the coordinating center and works under the guidance and leadership of the IHS Division of Diabetes Treatment and Prevention to provide technical assistance and collect, manage, and analyze performance data.

From 2006 to the present, each SDPI–DP project site has been instructed to 1) recruit and obtain informed consent from 48 AI/AN adults each year; 2) deliver the DPP’s 16-session Lifestyle Balance curriculum (3); 3) collect clinical data and responses to an 89-item questionnaire on sociodemographic characteristics, food choices, physical activity patterns, alcohol and tobacco use, and a range of psychosocial characteristics, at baseline, post-curriculum, and at annual intervals; and 4) send participant data to the coordinating center bimonthly.

Eligible AI/AN participants were aged 18 years or older and identified as at risk for diabetes by having diagnosed prediabetes (ie, an FBG of 100–125 mg/dL and a 2-hour oral glucose-tolerance test [OGTT] result of <200 mg/dL) and/or impaired glucose tolerance (ie, a 2-hour OGTT result of 140–199 mg/dL 2 hours after a 75-g oral glucose load and a FBG level <126 mg/dL). Exclusion criteria were 1) diagnosed diabetes, 2) pregnancy, 3) end-stage renal disease and dialysis, and 4) active alcohol or substance abuse, current cancer diagnosis, or other condition identified by a provider as a contraindication to participation.

### Intervention

Lifestyle Balance is a goal-based curriculum consisting of 16 educational sessions plus lifestyle coaching. Five of 16 sessions address healthy food choices and food preparation techniques designed to reduce fat and calorie intake. Eleven sessions cover physical activity, stress management, and self-motivation. A key aspect of the program is lifestyle coaches who deliver the curriculum one-on-one or in small groups to help participants achieve and maintain weight and behavior goals. Sessions last 30 to 60 minutes and are held in a community setting. Incentives (≤$30 in value) for completing the 16 sessions vary by program. The intervention is detailed elsewhere (13).

Intervention sites were allowed some flexibility in adapting the DPP strategies to fit the needs of their participants (14). The most frequent adaptations were group classes in nonclinical settings, information on local walking routes, and suggestions for modifying food preparation to reduce fat or increase fiber content of local dishes.

### Measures

Data were collected from January 2006 through July 2010. Baseline data on sociodemographic characteristics and diabetes-related factors were collected for participants who enrolled from January 2006 to July 2009. Participants underwent a medical examination to assess FBG, systolic and diastolic blood pressure (SBP and DBP), body mass index (BMI), and lipid levels (LDL, HDL, and triglycerides) at baseline, after completion of the Lifestyle Balance sessions (approximately 4–6 months after baseline, or “post-curriculum” hereafter), and then annually. Participants completed a survey on sociodemographic characteristics and health behaviors at each assessment.

Physical activity behaviors were assessed by using the Rapid Assessment of Physical Activity (RAPA), a 9-item instrument with response options of yes or no to questions on a range of weekly physical activity levels, strength training, and flexibility (15). A RAPA1 score was generated to measure participants’ weekly engagement in aerobic activity (scored from 1 [not engaged, sedentary] to 5 [regular activity]). A RAPA2 score was generated to measure engagement in strength training and flexibility activities (scored from 0 [engaged in neither], 1 [engaged in one but not both], or 2 [engaged in both].

Participants’ intake of 27 food items during the previous 30 days was assessed through a self-administered food frequency questionnaire adapted from the National Cancer Institute’s Multifactor Screener (16, 17). The intent of the food frequency questionnaire was to track changes in food choices, not to determine portion size or total nutrient intake. Frequency for each food type was reported as 1) less than once per month, 2) 1 to 3 times per month, 3) about once per week, 4) 2 or 3 times per week, 5) about once per day, and 6) more than once per day. Validation studies of short dietary assessment instruments indicate that these screeners are useful for characterizing a population’s median intakes, for distinguishing levels of intake (high or low) among individuals or groups, for tracking dietary changes among individuals or groups over time, and for examining interrelationships between diet and other variables (17).

For the SPDI-DP, some food-item questions were culturally modified or added through consultation with the programs. The processed meat question was expanded to include Spam (Hormel Foods Corporation) and corned beef. Corn tortillas, fry bread (deep-fried wheat-flour dough), other fried pastries, piñon nuts, and sunflower seeds were added to the food list. A composite foods query was added to include menudo (pork or beef stomach and red chili), guysava (roasted ground corn, beef, and chili), red and green chili, Indian tacos (ground beef, beans, and fry bread), dried corn soup, and wild rice soup. At the request of programs, a general question on “foods traditional to your tribe [specify]” was added. Although programs agreed that answers to this query would probably be inconsistent across tribes, the responses for each tribe would be shared with all programs. On the basis of these responses, some programs requested assistance with developing food inquiries specific to their tribe to collect information that might inform local education strategies. 

To evaluate overall patterns of food choice, we established 2 types of food scores. We first categorized the 27 food items as healthy (n = 6), not healthy (n = 12), or “undetermined” (n = 9). We created these categories by consensus in a survey of SDPI health educators. A healthy food score (α = 0.70) was constructed by averaging the frequency of consuming the 6 healthy foods, and an unhealthy food score (α = 0.74) was constructed by averaging the frequency of consuming the 12 unhealthy foods.

Study protocol was approved by the Colorado Multiple Institutional Review Board of the University of Colorado Anschutz Medical Center and the National IHS institutional review board. When required, grantees obtained approval from other entities charged with overseeing research in their programs (eg, tribal review boards, tribal councils). All participants provided written informed consent. 

### Statistical analysis

Differences in categorical sociodemographic characteristics between completers and noncompleters were examined by using χ^2^ tests. Completers were defined as those who completed all 3 assessments; noncompleters were defined as those who completed the baseline assessment but not both follow-up assessments. Differences in continuous variables (food scores, activity patterns, weight, BMI, and clinical measures) were examined by using 2-sample *t* tests. Paired *t* tests were used to test the significance of changes in the mean frequency of consumption of each food item and changes in healthy and unhealthy food score from baseline to post-curriculum and first annual assessment. The bivariable associations between changes in food scores and other diabetes-related factors (bodyweight, BMI, RAPA1, RAPA2, FBG, SBP, DBP, LDL, HDL and triglycerides) were examined using simple linear regression, with change in food score as the dependent variable and change in one of the diabetes-related factors as the independent variable in each model. Multiple linear regression was used to further assess the relationship between changes in food scores and diabetes-related factors while controlling for all diabetes-related factors and sociodemographic variables (sex, age, education, and income) in one model. Because fewer than 3% of participants self-identified as students, data on students were combined for analysis with data on unemployed participants. All analyses were conducted using SAS 9.2 software (SAS Institute Inc). Results were considered significant when *P* was .05 or less.

## Results

Most (74.3%) of the 3,135 participants who completed the baseline questionnaire (Table 1) were women, aged 40 or older (70.3%), had completed some college or were college graduates (63.2%), were employed (71.2%), had an annual household income of $30,000 or more (56.6%), and were married or living with a partner (57.9%). 

**Table 1:** Baseline Characteristics of Participants in Special Diabetes Program for Indians–Diabetes Prevention Demonstration Project, January 2006 to July 2010^a^^,^ b

Characteristic	All Participants (N = 3,135)	Completed Post-Curriculum^c^ Questionnaire (n = 2,046)	Did Not Complete Post-Curriculum^c^ Questionnaire (n = 1, 089)	*P* d	Completed First Annual Questionnaire (n = 1,480)	Did Not Complete First Annual Questionnaire (n = 1,655)	*P* d
**Sex**							
Male	805 (25.7)	503 (24.8)	302 (27.2)	.15	361 (24.7)	444 (26.6)	.22
Female	2,330 (74.3)	1,522 (75.2)	808 (72.8)		1,103 (75.3)	1,227 (73.4)	
**Baseline age group, y**							
18 to <40	932 (29.7)	524 (25.9)	406 (36.6)	<.001	350 (23.9)	580 (34.7)	<.001
40 to <50	913 (29.1)	608 (30.0)	303 (27.3)		450 (30.7)	461 (27.6)	
50 to <60	792 (25.3)	535 (26.4)	260 (23.4)		388 (26.5)	407 (24.4)	
≥60	498 (15.9)	358 (17.7)	141 (12.7)		276 (18.9)	223 (13.4)	
**Education**							
<High school	449 (15.2)	256 (13.3)	193 (18.8)	<.001	190 (13.5)	259 (16.7)	<.001
High school graduate	641 (21.7)	412 (21.3)	229 (22.3)		288 (20.5)	353 (22.8)	
Some college	1,330 (45.0)	869 (45.0)	461 (44.9)		638 (45.3)	692 (44.7)	
≥College graduate	538 (18.2)	395 (20.5)	143 (13.9)		292 (20.7)	246 (15.9)	
**Employment status**							
Employed	2,091 (71.2)	1,421 (74.2)	670 (65.5)	<.001	1,041 (74.5)	1,050 (68.2)	<.001
Retired	205 (7.0)	147 (7.7)	58 (5.7)		118 (8.4)	87 (5.7)	
Unemployed/student	642 (21.9)	347 (18.1)	295 (28.8)		239 (17.1)	403 (26.2)	
**Annual household income, $**							
<15,000	539 (21.4)	300 (18.2)	239 (27.7)	<.001	209 (17.3)	330 (25.2)	<.001
15,000 to <30,000	551 (21.9)	364 (22.0)	187 (21.6)		265 (21.9)	286 (21.9)	
30,000 to <50,000	721 (28.6)	494 (29.9)	227 (26.3)		364 (30.1)	357 (27.3)	
≥50,000	706 (28.0)	495 (30.0)	211 (24.4)		371 (30.7)	335 (25.6)	
**Marital status**							
Married or live with partner	1,532 (57.9)	1,041 (60.1)	491 (53.6)	.004	758 (60.3)	774 (55.6)	.006
Separated, divorced, or widowed	666 (25.2)	418 (24.1)	248 (27.1)		315 (25.1)	351 (26.3)	
Never married	450 (17.0)	273 (15.8)	177 (19.3)		184 (14.6)	266 (19.1)	
**Diabetes-related factors**							
Healthy food score^e^	3.4 (0.8)	3.4 (0.8)	3.4 (0.8)	.86	3.4 (0.8)	3.4 (0.8)	.61
Unhealthy food score^f^	2.9 (0.7)	2.8 (0.7)	3.0 (0.7)	<.001	2.8 (0.7)	2.9 (0.7)	<.001
Weight, lb	217.8 (52.4)	216.1 (52.2)	221.0 (52.6)	.01	215.1 (52.1)	220.1 (52.5)	.008
BMI	35.8 (7.5)	35.6 (7.5)	36.2 (7.4)	.04	35.5 (7.4)	36.1 (7.4)	.03
Minutes of physical activity/week	99.9 (192.4)	97.1 (176.5)	104.9 (218.4)	.31	98.5 (181.8)	101.0 (91.3)	.71
RAPA1^g^	3.8 (1.1)	3.8 (1.1)	3.7 (1.1)	.27	3.8 (1.1)	3.7 (1.1)	.009
RAPA2^h^	0.7 (1.1)	0.7 (1.1)	0.8 (1.1)	.64	0.7 (1.1)	0.7 (1.1)	.71
FBG, mg/dL	104.6 (9.3)	104.4 (9.0)	104.9 (9.7)	.17	104.2 (9.1)	104.9 (9.5)	.06
SBP, mm Hg	126.2 (14.9)	126.3 (14.5)	126.0 (15.4)	.48	126.6 (14.6)	125.9 (15.1)	.20
DBP, mm Hg	78.3 (10.2)	78.5 (9.91)	78.1 (10.7)	.33	78.7 (9.7)	78.0 (10.6)	.03
LDL, mg/dL	110.9 (31.0)	111.4 (30.7)	110.0 (31.4)	.22	111.8 (31.2)	110.1 (30.8)	.12
HDL, mg/dL	45.1 (11.9)	45.3 (12.2)	44.8 (11.4)	.26	45.3 (11.9)	44.9 (12.0)	.40
Triglycerides, every 10 mg/dL	160.7 (98.3)	162.6 (98.2)	157.1 (98.4)	.14	163.7 (101.4)	158.0 (95.4)	.11

Abbreviations: BMI, body mass index; DBP, diastolic blood pressure; DPP, Diabetes Prevention Program; FBG, fasting blood glucose; HDL, high-density lipoprotein; LDL, low-density lipoprotein; RAPA1, Rapid Assessment of Physical Activity: Aerobic; RAPA2, Rapid Assessment of Physical Activity: Strength and Flexibility; SBP, systolic blood pressure.

a All values are mean (percentage) unless otherwise indicated.

b Baseline data were collected for participants who enrolled from January 2006 to July 2009.

c After the DPP’s 16-session Lifestyle Balance curriculum.

d *P* values determined by using χ^2^ test.

e Constructed by averaging the frequency of consuming 6 healthy foods. Scored from 1 to 6 with 1 = less than once per month; 2 = 1 to 3 times per month; 3 = about once per week; 4 = 2 or 3 times per week; 5 = about once per day; and 6 = more than once per day.

f Constructed by averaging the frequency of consuming 12 unhealthy foods. Scored from 1 to 6 with 1 = less than once per month; 2 = 1 to 3 times per month; 3 = about once per week; 4 = 2 or 3 times per week; 5 = about once per day; and 6 = more than once per day.

g Scored as weekly engagement in aerobic activity from 1 (not engaged, sedentary) to 5 (regular activity).

h Scored as 0, 1, or 2 to measure engagement in strength training and flexibility activities: 0 (engaged in neither), 1 (engaged in one but not both), or 2 (engaged in both).

Of the 3,135 baseline participants, 65.3% (n = 2,046) completed the post-curriculum questionnaire, and 47.2% (n = 1,480) finished the first annual questionnaire. Compared with completers at baseline, noncompleters were younger, had less formal education, lived in lower-income households, were less likely to be married or living with a partner, and were more likely to be unemployed, eat unhealthy foods, and have a higher bodyweight and BMI; noncompleters also had lower DBP and lower RAPA1 scores at the first annual questionnaire.

Intake of all 6 healthy food items and mean healthy food score significantly increased post-curriculum and at first annual assessment from baseline (Table 2). Correspondingly, post-curriculum intake of all unhealthy food items (except 100% fruit juice) and mean unhealthy food score decreased from baseline. At first annual assessment, participants still had a lower intake of unhealthy foods (except 100% fruit juice) than they did at baseline.

**Table 2:** Intake Frequency^a^ of Food Items^b^ at Baseline, Post-Curriculum^c^, and First Annual Assessment Among Participants in the Special Diabetes Program for Indians–Diabetes Prevention Demonstration Project, January 2006 to July 2010^d^

Food Items	Baseline (N = 3,135)	Baseline Paired With Post-Curriculum Assessment (n = 2,046)	Post-Curriculum (n = 2,046)	*P^e^*	Baseline Paired With First Annual Assessment (n = 1,480)	First Annual Assessment (n = 1,480)	*P^e^*
**Healthy foods**							
Whole grain bread	3.7 (1.6)	3.8 (1.5)	4.2 (1.4)	<.001	3.8 (1.6)	4.0 (1.4)	<.001
Fruit	3.7 (1.4)	3.8 (1.4)	4.3 (1.3)	<.001	3.8 (1.4)	4.1 (1.4)	<.001
Green leafy salad	3.3 (1.3)	3.2 (1.3)	3.7 (1.2)	<.001	3.3 (1.3)	3.6 (1.2)	<.001
Cooked dried beans	2.2 (1.1)	2.2 (1.0)	2.4 (1.1)	<.001	2.2 (1.0)	2.3 (1.1)	<.001
Fish/chicken/game	3.4 (1.1)	3.5 (1.1)	3.6 (1.1)	<.001	3.5 (1.1)	3.6 (1.1)	.002
Vegetables	4.0 (1.3)	4.0 (1.2)	4.4 (1.2)	<.001	4.0 (1.3)	4.2 (1.2)	<.001
Mean score for all healthy foods	3.4 (0.8)	3.4 (0.8)	3.8 (0.8)	<.001	3.4 (0.8)	3.6 (0.8)	<.001
**Unhealthy foods**							
Bacon or sausage	2.5 (1.1)	2.5 (1.1)	2.1 (1.0)	<.001	2.5 (1.1)	2.2 (1.1)	<.001
Processed meat	2.8 (1.3)	2.8 (1.3)	2.3 (1.2)	<.001	2.7 (1.2)	2.4 (1.2)	<.001
Processed flour	3.2 (1.5)	3.2 (1.5)	2.6 (1.4)	<.001	3.2 (1.6)	2.9 (1.4)	<.001
Fry bread^f^ or pastries	1.8 (1.0)	1.8 (1.0)	1.5 (0.8)	<.001	1.8 (1.0)	1.6 (0.8)	<.001
Baked goods	2.6 (1.2)	2.6 (1.2)	2.0 (1.0)	<.001	2.5 (1.2)	2.1 (1.0)	<.001
Soft drinks	3.1 (1.9)	3.0 (1.8)	2.3 (1.6)	<.001	3.0 (1.8)	2.5 (1.6)	<.001
100% Fruit juice	2.9 (1.5)	2.8 (1.5)	2.9 (1.5)	.08	2.8 (1.5)	2.8 (1.5)	.44
Add sugar or creamer to coffee or tea	3.5 (2.0)	3.5 (2.1)	3.0 (2.0)	<.001	3.5 (2.1)	3.2 (2.1)	<.001
Regular salad dressing or mayonnaise	2.9 (1.4)	2.9 (1.3)	2.2 (1.3)	<.001	2.9 (1.3)	2.4 (1.3)	<.001
Fried potatoes	2.9 (1.2)	2.9 (1.2)	2.1 (1.1)	<.001	2.8 (1.2)	2.4 (1.1)	<.001
Red meat	3.4 (1.3)	3.4 (1.3)	3.1 (1.3)	<.001	3.4 (1.3)	3.3 (1.3)	.04
Fast food	3.0 (1.2)	3.0 (1.2)	2.3 (1.1)	<.001	2.9 (1.2)	2.5 (1.1)	<.001
Mean score for all unhealthy foods	2.9 (0.7)	2.8 (0.7)	2.4 (0.7)	<.001	2.8 (0.7)	2.5 (0.7)	<.001
**Items of undetermined healthfulness**							
Cereal	3.1 (1.4)	3.1 (1.4)	3.6 (1.4)	<.001	3.1 (1.4)	3.4 (1.4)	<.001
Coffee or tea	4.4 (1.8)	4.4 (1.8)	4.2 (1.9)	<.001	4.5 (1.8)	4.4 (1.8)	.22
Other white potatoes	2.9 (1.1)	2.9 (1.1)	2.7 (1.1)	<.001	2.9 (1.1)	2.7 (1.1)	<.001
Pasta	3.3 (1.1)	3.2 (1.1)	3.0 (1.1)	<.001	3.2 (1.1)	3.2 (1.1)	.06
Nuts or seeds	2.6 (1.4)	2.6 (1.4)	2.4 (1.3)	<.001	2.6 (1.4)	2.5 (1.3)	<.001
Snacks or junk food	3.2 (1.3)	3.2 (1.3)	2.4 (1.2)	<.001	3.2 (1.3)	2.6 (1.2)	<.001
Soups or stews	1.8 (1.0)	1.8 (1.0)	1.6 (0.9)	<.001	1.8 (1.0)	1.7 (0.9)	.006
Milk	3.5 (1.6)	3.5 (1.6)	3.6 (1.6)	<.001	3.5 (1.6)	3.6 (1.6)	.02
Foods traditional to tribe	1.9 (1.2)	1.9 (1.2)	1.8 (1.1)	<.001	1.9 (1.2)	1.8 (1.1)	.07
Mean score for all undetermined items	3.0 (0.7)	3.0 (0.6)	2.8 (0.6)	<.001	3.0 (0.6)	2.9 (0.6)	<.001

Abbreviation: DPP, Diabetes Prevention Program.

a Scored from 1 to 6 with 1 = less than once per month; 2 = 1 to 3 times per month; 3 = about once per week; 4 = 2 or 3 times per week; 5 = about once per day; and 6 = more than once per day.

b Categories of food items (healthy, unhealthy, undetermined) were established by consensus in a survey of Special Diabetes Program for Indians health educators.

c After DPP’s 16-session Lifestyle Balance curriculum.

d All values are mean (standard deviation) unless otherwise indicated.

e *P* values determined by 2-sample* t *tests.

f Deep-fried wheat-flour dough.

Post-curriculum and at first annual assessment, a reduction in bodyweight and BMI was associated with an increased healthy food score and decreased unhealthy food score (Table 3). Associations between changes in healthy food scores and unhealthy food scores and changes in activity patterns were also evident. Regular engagement in aerobic activity (RAPA1) and in flexibility and strength training activities (RAPA2) were associated with an increased healthy food score and a decreased unhealthy food score post-curriculum and at first annual assessment.

**Table 3:** Bivariable Associations Between Change in Diabetes-Related Factors and Change in Food Scores Among Participants in the Special Diabetes Program for Indians–Diabetes Prevention Demonstration Project, January 2006 to July 2010

Decrease in Diabetes-Related Factor	Post-Curriculum Assessment Minus Baseline				First Annual Assessment Minus Baseline			
	Change in Healthy Food Score^a^		Change in Unhealthy Food Score^b^		Change in Healthy Food Score^a^		Change in Unhealthy Food Score^b^	
	β	*P* c	β	*P* c	β	*P* c	β	*P* c
Weight, every 10 lb	0.105	<.001	−0.124	<.001	0.035	.01	−0.101	<.001
BMI, every 10 points	0.600	<.001	−0.738	<.001	0.196	.02	−0.614	<.001
RAPA1, every 1 point	−0.108	<.001	0.099	<.001	−0.078	<.001	0.055	<.001
RAPA2, every 1 point	−0.073	<.001	0.053	<.001	−0.068	<.001	0.030	.03
FBG, every 10 mg/dL	0.049	.002	−0.047	.001	0.004	.81	−0.039	.004
SBP, every 10 mm Hg	0.006	.58	−0.016	.11	0.001	.91	−0.021	.0497
DBP, every 10 mm Hg	0.030	.06	−0.019	.19	0.012	.49	−0.006	.70
LDL, every 10 mg/dL	0.025	<.001	−0.005	.41	0.007	.41	−0.016	.03
HDL, every 10 mg/dL	−0.035	.09	−0.036	.06	−0.036	.11	−0.008	.70
Triglyceride, every 10 mg/dL	0.007	<.001	−0.004	.03	−0.002	.50	−0.004	.04

Abbreviations: BMI, body mass index; DPP, Diabetes Prevention Program; FBG, fasting blood glucose; HDL, high-density lipoprotein; LDL, low-density lipoprotein; RAPA1, Rapid Assessment of Physical Activity: Aerobic; RAPA2, Rapid Assessment of Physical Activity: Strength and Flexibility; SBP, systolic blood pressure.

a Constructed by averaging the frequency of consuming 6 healthy foods. Scored from 1 to 6 with 1 = less than once per month; 2 = 1 to 3 times per month; 3 = about once per week; 4 = 2 or 3 times per week; 5 = about once per day; and 6 = more than once per day.

b Constructed by averaging the frequency of consuming 12 unhealthy foods. Scored from 1 to 6 with 1 = less than once per month; 2 = 1 to 3 times per month; 3 = about once per week; 4 = 2 or 3 times per week; 5 = about once per day; and 6 = more than once per day.

c *P* values determined using simple linear regression.

From baseline to post-curriculum, decreased FBG was associated with an increased healthy food score and decreased unhealthy food score. From baseline to the first annual assessment, the negative association between FBG and a healthy food score was not maintained, but the positive association with the unhealthy food score was still evident. A decrease in triglycerides and LDL was associated with an increased healthy food score post-curriculum but not at the first annual assessment. A decrease in triglycerides was associated consistently with a decreased unhealthy food score at post-curriculum and at first annual assessment. No consistent significant relationships were observed for SBP, DBP, or HDL with food scores post-curriculum or at first annual assessment.

When we controlled for other variables in the model, BMI and RAPA1 showed strong associations with changes in food scores (Table 4). Reduced BMI was associated with increased healthy food choices post-curriculum and decreased unhealthy food choices both post-curriculum and at first annual assessment. Greater regularity of aerobic activity was associated with increased healthy food choices and decreased unhealthy food choices both post-curriculum and at first annual assessment. In addition, reduced LDL was significantly associated with increased healthy food choices post-curriculum but not at first annual assessment. When we controlled for other variables in the models, changes in FBG, SBP, HDL, and triglycerides were no longer associated with changes in food scores.

**Table 4:** Multivariable Associations Between Change in Diabetes-Related Factors and Change in Food Scores Among Participants in Special Diabetes Program for Indians–Diabetes Prevention Demonstration Project^a^, January 2006 to July 2010

Decrease in Diabetes-Related Factor	Post-DPP Curriculum Assessment Minus Baseline				First Annual Assessment Minus Baseline			
	Change in Healthy Food Score^b^		Change in Unhealthy Food Score^c^		Change in Healthy Food Score^b^		Change in Unhealthy Food Score^c^	
	β	*P* d	β	*P* d	β	*P* d	β	*P* d
BMI, every 10 points	0.545	<.001	−0.677	<.001	0.161	.11	−0.571	<.001
RAPA1	−0.106	<.001	0.076	<.001	−0.089	<.001	0.036	.02
FBG, every 10 mg/dL	0.014	.46	−0.008	.63	−0.010	.60	−0.009	.59
SBP, every 10 mm Hg	−0.008	.51	−0.014	.19	−0.011	.40	−0.021	.07
LDL, every 10 mg/dL	0.017	.03	0.005	.47	0.001	.89	−0.008	.31
HDL, every 10 mg/dL	−0.030	.23	−0.039	.08	−0.039	.13	−0.035	.13
Triglycerides, every 10 mg/dL	0.002	.60	−0.0003	.92	−0.006	.10	−0.005	.10

Abbreviations: BMI, body mass index; DPP, Diabetes Prevention Program; FBG, fasting blood glucose; HDL, high-density lipoprotein; LDL, low-density lipoprotein; RAPA1, Rapid Assessment of Physical Activity: Aerobic; SBP, systolic blood pressure.

a Controlling for sociodemographic characteristics and other diabetes risk factors.

b Constructed by averaging the frequency of consuming 6 healthy foods. Scored from 1 to 6 with 1 = less than once per month; 2 = 1 to 3 times per month; 3 = about once per week; 4 = 2 or 3 times per week; 5 = about once per day; and 6 = more than once per day.

c Constructed by averaging the frequency of consuming 12 unhealthy foods. Scored from 1 to 6 with 1 = less than once per month; 2 = 1 to 3 times per month; 3 = about once per week; 4 = 2 or 3 times per week; 5 = about once per day; and 6 = more than once per day.

d *P* values determined using multiple linear regression.

## Discussion

SDPI-DP outcomes provide a national picture of the impact of food choice on clinical diabetes-related factors among AI/AN adults with prediabetes living in diverse geographic community settings who desired to decrease their risk of diabetes. At baseline, SPDI-DP participants’ food choices were similar to the choices of national populations (ie, regular intake of red meat, processed wheat-flour baked goods, soft drinks, fried foods); these choices contribute to a high-fat, low-fiber diet (18). After the Lifestyle Balance curriculum and lifestyle coaching, program participants increased their intake of healthy foods (ie, whole grains, low-fat meats, fruits, and vegetables) and significantly decreased their intake of unhealthy foods, most notably high-fat meats, baked goods and pastries, soft drinks, fried potatoes, and fast foods. The extent and persistence of the food choice changes and the associations with clinical and other behavioral diabetes-related factors varied over time. Immediately post-curriculum, an increase in healthy food score was associated with reductions in BMI, FBG, LDL, and triglycerides and an increase in aerobic, strength, and flexibility activities. At first annual assessment, the only associations that persisted were between both food scores and aerobic activity (RAPA1) and unhealthy food score and BMI.

The lack of associations between food scores and SBP, DBP, and HDL may reflect the well-documented observation that blood pressure is significantly influenced by other factors, such as smoking status, salt intake, and psychological distress (19–21). HDL is also influenced by nonfood-related factors and can be particularly slow to change (7).

Long-term follow-up in the Finnish Diabetes Prevention Study (22) and the Chinese Da Qing Diabetes Prevention Study (23) demonstrates that lifestyle-behavior changes can have a sustained impact and are more cost-effective than oral hypoglycemic drugs (7). The selection of healthy foods at the first annual assessment in the SDPI–DP project demonstrates the sustainability of behavior change and shows that the DPP strategy is effective among AI/ANs with diagnosed prediabetes. The SDPI–DP project participants had other health improvements, such as substantial weight loss and increased physical activity; these improvements were similar to those found the original DPP trial (14).

Lifestyle interventions are effective in high-resource settings (eg, those with recreational facilities and grocery stores with abundant fresh food selections), but evidence is needed to demonstrate the health benefits of such interventions in low-resource settings and high-risk populations (24,25). In the SDPI-DP, a key component of lifestyle modification (healthful dietary change) was achieved and maintained among AI/ANs from diverse community settings, further illustrating that modest changes in food choices yield modest reductions in weight, which in turn translates to significant reductions in diabetes risk. A review of how each site tailored the curriculum and lifestyle coaching to address local food resources and preferences would expand our understanding of how local knowledge can enhance the effectiveness of evidence-based health promotion strategies.

The challenge that remains is motivating and retaining young, less educated, unemployed, and low-income AI/ANs who did not complete the Lifestyle Balance curriculum. The educational sessions in our program were most often offered in group settings. The delivery format may not have appealed to young adults who are more comfortable with online learning; class times may have conflicted with job-hunting activities or other commitments; most sites provided child care to address potential conflicts with parenting responsibilities. Reaching young adults at risk for diabetes may require innovative teaching strategies that use digital technology and support independent learning.

This analysis of a SDPI-DP data set has several limitations. Data on food intake and physical activity were self-reported and are subject to social desirability and recall bias. Project participation was voluntary; thus the sample comprises people interested in improving their health. Participants who dropped out were more likely to be at greater diabetes risk at baseline than those who completed the program; they had a higher BMI, greater intake of unhealthy foods, and less education. This may imply potential “survivor bias”: only outcomes of those motivated to reduce diabetes risk through this intervention approach were recorded and analyzed. Although development of the food frequency questionnaire was guided by extensive program consultation, the heterogeneity of AI/ANs nationally limits its specificity. Food items and descriptions were driven by the regional diversity of the 36 programs and may have been interpreted in various ways by participants. Also, the questionnaire was adapted from the National Cancer Institute’s Multifactor Screener, a tool intended to provide an estimate of usual intake. It was reviewed by health professionals at each of the 36 sites for comprehension and relevance, but it was not validated through another method of dietary data collection. However, the significant associations between changes in healthy food choices and improved physical activity levels as well as other clinical diabetes-related factors provide strong support for the instrument’s criterion validity. Finally, the results were used only to assess food choice and not nutrient intake.

AI/AN adults participating in a community-based implementation of the DPP-based lifestyle intervention made and maintained healthy food choices both immediately post-intervention and at the first annual assessment. These changes in food choices were associated with significant and clinically meaningful improvements in diabetes-related factors. The SDPI–DP project demonstrated that local implementation of this evidence-based strategy can yield sustained dietary change and reductions in diabetes risk factors among AI/AN adults with diagnosed prediabetes. These findings, from a national initiative involving AI/AN participants from diverse cultural and geographic settings, advance our understanding of the role that food choice plays in reducing diabetes risk factors in a population that has been disproportionately affected by this chronic, disabling disease.
